# Can We Depend on Investigators to Identify and Register Randomized Controlled Trials?

**DOI:** 10.1371/journal.pone.0044183

**Published:** 2012-09-11

**Authors:** Roberta W. Scherer, Pamela C. Sieving, Ann-Margret Ervin, Kay Dickersin

**Affiliations:** 1 Johns Hopkins Bloomberg School of Public Health, Baltimore, Maryland, United States of America; 2 National Institutes of Health Library, National Institutes of Health, Bethesda, Maryland, United States of America

## Abstract

**Purpose:**

To reduce publication bias, systematic reviewers are advised to search conference abstracts to identify randomized controlled trials (RCTs) conducted in humans and not published in full. We assessed the information provided by authors to aid identification of RCTs for reviews.

**Methods:**

We handsearched the Association for Research in Vision and Ophthalmology (ARVO) meeting abstracts for 2004 to 2009 to identify reports of RCTs. We compared our classification with that of authors (requested by ARVO 2004–2006), and authors’ report of trial registration (required by ARVO 2007–2009).

**Results:**

Authors identified their study as a clinical trial for 169/191 (88%; 95% CI, 84–93) RCTs we identified for 2004, 174/212 (82%; 95% CI, 77–87) for 2005 and 162/215 (75%; 95% CI, 70–81) for 2006. Authors provided registration information for 107/172 (62%; 95% CI, 55–69) RCTs for 2007, 103/153 (67%; 95% CI, 60–75) for 2008, and 126/171 (74%; 95% CI, 67–80) for 2009. Most RCT authors providing a trial register name specified ClinicalTrials.gov (276/312; 88%; 95% CI, 85–92) and provided a valid ClinicalTrials.gov registration number (261/276; 95%; 95% CI, 92–97). Based on information provided by authors, trial registration information would be accessible for 48% (83/172) (95% CI, 41–56) of all ARVO abstracts describing RCTs in 2007, 63% (96/153) (95% CI, 55–70) in 2008, and 70% in 2009 (118/171) (95% CI, 62–76).

**Conclusions:**

Authors of abstracts describing RCTs frequently did not classify them as clinical trials nor comply with reporting trial registration information, as required by the conference organizers. Systematic reviewers cannot rely on authors to identify relevant unpublished trials or report trial registration, if present.

## Introduction

Randomized controlled trials (RCTs) that are used to inform systematic reviews are typically identified through searching electronic databases, such as PubMed, EMBASE, and the Cochrane Central Register of Controlled Clinical Trials. Because only 60% of trials initially reported as conference abstracts are ever published in full [1], systematic review authors who neglect to search the grey literature, especially conference abstracts, may fail to identify relevant unpublished trials. Further, because of publication bias, the tendency to publish results based on the strength or direction of findings, systematic reviews that focus only on trials reported in full may present a biased representation of existing evidence.

Clinical trial registration was proposed in the 70’s and 80’s, in part as a mechanism of combating reporting biases [2.3], and the notion slowly gained ground in the 90’s and 2000’s [4], [5], [6], especially following support from the International Committee of Medical Journal Editors (ICMJE)[7]. Today, registration has been resoundingly endorsed by the scientific community [8], [9], and systematic review authors are examining the potential for making use of register information to complement what is available in the published literature [10].

With 100 to 200 abstracts describing clinical trials published annually, the Association for Research in Vision and Ophthalmology (ARVO) annual meeting is an important source of reports of RCTs for the Cochrane Eyes and Vision Group (CEVG). We have searched the ARVO meeting books or the ARVO online abstract repository from 1990 forward for reports of RCTs and controlled clinical trials, to contribute to CEVG’s specialized database of trial reports. Because clinical trials represent only 2 to 3% of ARVO abstracts published each year, and completing the searches requires about 45 hours per year [11], we began a project in 2004 with the initial objective of determining whether it is possible to develop a valid and reliable system for author classification of clinical trials. If so, such a system would obviate the need for labor-intensive handsearching. In response to our request, ARVO conference organizers added a definition and check-off box to the 2004 abstract submission form to indicate whether the submitted abstract described a “human clinical trial”. The conference organizers revised the check-off box in 2005 and 2006 by asking authors if the submitted abstract described a “human controlled clinical trial”. The information included on the ARVO webpage for 2004 and 2005 included the following:

**2004 Question on the Abstract Submission Form:**

“Is the research presented in your abstract a human clinical trial? [Yes] [No] (see hyperlink definition)

**Hyperlink definition of a human clinical trial:** *“a planned study in humans designed to assess the efficacy and/or safety of one or more test interventions by comparing outcomes in individuals assigned the test intervention(s) with those receiving no intervention or a comparison intervention, and where individuals in all groups are enrolled, treated, and followed concurrently.”*

**2005 and 2006 Question on the Abstract Submission Form:**

“Is the research presented in your abstract a human controlled clinical trial?” [Yes] [No] (same hyperlink definition as 2004)

In 2005, ARVO adopted a policy requiring registration of controlled trials in an electronically searchable, publicly available register before submission of abstracts to the ARVO annual meeting or articles to the associated journal, *Investigative Ophthalmology & Visual Science* [12]. Thus, starting in 2007, meeting organizers removed the clinical trials check box from the annual meeting Abstract Submission Form, and replaced the box with a field to designate the name of a trial register and the trial registration number. The information included on the ARVO webpage from 2007 through 2009 included the following information:

**2007: Clinical Trial Registration**

“Please answer the following information below regarding Clinical Trials Registration. (Required)

Does the research presented in your abstract report on a clinical trial (refer to item #4 of the “ARVO Statement on Registering Clinical Trials” [hyperlink] and/or FAQs [hyperlink]) about the ARVO policy. [Yes] [No]”

Hyperlink to item#4 “*a* ***“clinical trial”*** *consists of any study involving a new therapy of any kind, whether medical, surgical, psychological, or sociological, in which subjects are concurrently divided into two or more groups. …]*

A hyperlink to “Clinical Trial Explanation” was also included.

**2008 Clinical Trial Registration** The Abstract Submission Form was the same as in 2007, expect that the hyperlink to “Clinical Trial Explanation” was deleted and a drop-down menu of trial registers was added that included the following:

www.actr.org.au

www.clinicaltrials.gov

www.ISRCTN.org

www.umin.ac.jp/ctr/index/htm

www.trialregister.nl/trialreg/index.asp

**2009 Clinical Trial Registration**

“Please answer the following information below regarding Clinical Trial Registration. (Required)

Clinical trials require registration with a publicly accessible clinical trials registry that is approved by the World Health Organization (WHO). All abstracts that describe results from a clinical trial must include the registry site and registration number of the trial. To determine if the study results presented in your abstract are from a clinical trial, consider the following questions and refer to the “ARVO Statement on Registering Clinical Trials” [hyperlink] and/or FAQs [hyperlink]) about the ARVO policy.

1. Does this study involve a therapeutic intervention in human subjects? (The intervention may be of any kind, e.g., medical, surgical/laser, or psychological/sociological.)

2. Is the study prospective?

If the answer is “No” to either question, then the study does not meet the current definition of a clinical trial, and does not need to be registered. Select “No” below. If the answer is “Yes” to both questions, then the study does meet the definition of a clinical trial, regardless of the number of subjects involved or whether it involves comparison groups (i.e., different doses of a drug, or treatment and control groups) and must be registered. Select “Yes” below, then select the registry site and enter the corresponding registration number.

*Does the study meet the definition of a clinical trial? [Yes] [No]”

The drop-down was revised to include the following trial registers:

www.anzctr.org.au

www.clinicaltrials.gov

www.ISRCTN.org

www.chictr.org

www.ctri.in

www.umin.ac.jp/ctr/index/htm

www.slctr.lk

www.trialregister.nl/trialreg/index.asp

Our overall study objective was to assess the reliability of information provided by ARVO abstract authors that might be used to aid in identification of relevant abstracts for systematic reviews. We evaluated whether authors correctly classify abstracts describing RCTs as clinical trials, and as an adjunct to this, determined where investigators are registering their RCTs.

## Methods

### Identification of Randomized Controlled Clinical Trials

We searched the ARVO annual meeting abstract book (in 2004 we searched a CD-ROM; in 2005 through 2009 we searched online at http://www.arvo.org) to identify abstracts describing RCTs and controlled clinical trials for inclusion in the CEVG specialized trial register. For this study, we include only abstracts reporting RCT findings. Abstracts were classified as RCTs if “individuals (or other units) followed in the trial were assigned prospectively to one of two (or more) alternative forms of health care using random allocation” [13]. Because the original purpose of our handsearch of the abstracts was to identify RCTs for the CEVG trial register, and reviewing all abstracts for each year is labor intensive, a single individual (RWS) completed the initial handsearch; this was done in the year following each annual meeting. We downloaded and printed a hard copy of each abstract classified as an RCT.

### Author Classification of Abstract as a Trial

Following each annual meeting, the meeting organizers provided us with an electronic list of abstract program numbers that were identified by the author(s) as a “human clinical trial” (2004) or “human controlled clinical trial” (2005 and 2006) report (see Box for definitions). For the 2007 meeting, the meeting organizers provided an electronic list of the program number, trial register name, and registration number for all abstracts with trial registration information. For the 2008 and 2009 meetings, the organizers provided a hardcopy lists that included this information. We searched for and obtained paper copies of the abstracts on the organizers’ lists that we had not already identified as RCTs (2004 through 2009).

### Reference Standard

We compared the list of abstracts provided by the meeting organizers with the abstracts that we had previously classified as RCTs. Any abstract on the list that the handsearcher had not classified as an RCT was re-evaluated. If the handsearcher agreed that the report described an RCT, then that abstract was included in the reference standard for that year.

Thus, for each year, there were two groups of studies:

Abstracts in the reference standard:

$ Classified as a clinical trial by the author and as an RCT by the handsearcher;
$ Not classified as a clinical trial by the author, but classified as an RCT by the handsearcher.

Abstracts not in the reference standard:

$ Classified as a clinical trial by the author, but not classified as an RCT the handsearcher.
$ Not classified as a clinical trial by the author, nor classified as an RCT by the handsearcher.

A second handsearcher (PCS) reviewed the classification of 100% of abstracts that were included in the reference standard, i.e., abstracts that were classified by the handsearcher as an RCT. A 10% sample of abstracts classified by the author as a clinical trial, but not as an RCT by the first handsearcher, and a 5% sample of abstracts not classified as a clinical trial by the author nor as an RCT by the handsearcher were reviewed by another handsearcher (KD or AE). Abstracts in question were re-reviewed by the lead author for a decision or by both readers to arrive at consensus.

### Characteristics of Trial Registration Information

We classified the type of organization recorded in the trial registration field (trial register, ethics board/institutional review board, regulatory agency, local authority, or other) for conference abstracts presented in 2007, 2008, and 2009. For trials in the reference standard that were reported by the author as registered at ClinicalTrials.gov, we verified the information by entering the trial registration number provided in the ClinicalTrials.gov search webpage [http://ClinicalTrials.gov].

### Data Analyses

We entered into an Access database the program number and year of presentation of each abstract describing an RCT that we identified by handsearching the conference books, and imported the program number and year of presentation received from the meeting organizers for the abstracts from the years 2004 to 2006 as well as the program number, year of presentation, register name, and registration number for abstracts presented in years 2007 to 2009. We calculated the proportion of RCTs in the total number of abstracts presented at the conference for 2005–2009, and the proportion of RCTs in our reference standard that were correctly identified by the author, performing separate comparison analyses by year. We tabulated information recorded in the trial register fields, including trial register “name” and valid trial register number (yes/no). Results are presented as point estimates with 95% confidence intervals (CI). Two individuals performed all calculations independently.

## Results

### Classification of Abstracts

The first handsearcher classified 1,121 of the abstracts presented at ARVO from 2004 to 2009 as RCTs. A second person read all 1,121 abstracts and classified 1,101 as RCTs, 10 as questionable, and 10 as “not RCT”. After re-review the 2 readers reached consensus on the 20 articles where there was initial disagreement;, resulting in 1,114 abstracts that were included in the reference standard as RCTs.

The first handsearcher classified 1529 abstracts, originally categorized by the author as a clinical trial, as “not RCTs”. A second person read a 10% sample (153) of these abstracts and classified 3 as RCTs and 150 as “not RCTs.” The first handsearcher, re-reviewed the 3 abstracts classified as RCTs by the second reader, and decided to keep the original classification of “not RCT.”

The first handsearcher classified 32,565 abstracts, which had not been classified as a clinical trial by the author, as “not RCT.” A second person read a 5% sample (1628 abstracts), and classified all as “not RCT.”

### Author Classification of Studies

The number of RCTs presented annually at the 2004 to 2009 ARVO meetings and in the reference standard ranged from 153 to 215. Authors identified 169/191 (88%; 95% CI, 84–93) of all RCTs we identified for 2004, 174/212 (82%; 95% CI, 77–87) for 2005, and 162/215 (75%; 95% CI, 70–81) for 2006, by checking the “human clinical trial” or “human controlled clinical trial” box. A lower proportion of RCTs were identified by authors in 2007 (107/172; 62%; 95% CI, 55–70), 2008 (103/153; 67%; 95% CI, 60–75), and 2009 (126/171; 74%; 95% CI, 67–80), years requiring trial registration information rather than a checked box to designate a clinical trial (see Table 1). However, the majority of studies that authors identified were of other types of study design rather than RCTs; only 841/2372 (35%; 95% CI, 34–37) abstracts classified as a clinical trial by authors described an RCT. The remaining abstracts described non-randomized controlled clinical trials, uncontrolled clinical trials, studies not involving humans, or trials in which participants were assigned to a treatment group based on some participant characteristic (e.g., severity of disease) (see Table 2).

**Table 1:** Reference standard RCTs identified and RCTS identified by author as controlled trial by year of presentation at ARVO.

Year of meeting	Reference standard RCTs	Reference standard RCTs classifiedby author as clinical trial	Abstracts classified by author as clinical trial	ARVO abstracts presented
	No.	No. (%)	No.	No.
**2004**	191	169 (88)	634*	5,610
**2005**	212	174 (82)	487†	5,732
**2006**	215	162 (74)	454†	5,920
**2007**	172	107 (62)	258‡	6,044
**2008**	153	103 (67)	252‡	6,122
**2009**	171	126 (74)	287‡	5,787§
**Total**	1,114	841 (75)	2,372	35,215

* Box checked “Yes” for “human clinical trial” (n = 634).

† Box checked “Yes” for “human controlled clinical trial” (n = 941).

‡ Information recorded in trial registration box (n = 797).

§ Does not include 576 abstracts that were withdrawn and are not included in the analyses.

**Table 2:** Abstracts identified by author as controlled trial by study design and year of presentation at ARVO.

Year	CEVG classification of abstracts identified by author as controlled trial						
	RCT	Non-randomized trial	Not human	Un-controlled case series	Comparison by group characteristic	Other§	Total
	No. (%)	No. (%)	No. (%)	No. (%)	No. (%)	No. (%)	No.
**2004** *	169 (26.7)	59 (9.3)	13 (2.0)	195 (30.8)	140 (22.1)	58 (9.1)	634
2005**	174 (35.7)	58 (11.9)	17 (3.5)	139 (28.5)	87 (17.9)	12 (2.5)	487
**2006** †	162 (35.6)	60 (13.2)	20 (4.4)	130 (28.6)	59 (13.0)	23 (5.1)	454
**2007** ‡	107 (41.5)	18 (7.0)	14 (5.4)	96 (37.2)	20 (7.8)	3 (1.2)	258
**2008** ‡	103 (40.9)	13 (4.6)	2 (0.8)	87 (34.5)	34 (13.5)	13 (5.2)	252
**2009** ‡	126 (43.9)	18 (6.3)	3 (1.0)	97 (33.8)	19 (616)	24 (8.4)	287
**Total**	841 (35.5)	226 (9.5)	69 (2.9)	744 (31.3)	359 (15.1)	133 (5.6)	2,372

* Box checked “Yes” for “human clinical trial”.

† Box checked “Yes” for “human controlled clinical trial”.

‡ Information recorded in trial registration box.

§ Includes: description of study methods (e.g., methods for measuring outcomes), systematic reviews, theoretical models, studies on correlation between test methods, and studies with historical controls).

### Registration of Clinical Trials

Authors provided trial registration information at abstract submission for 797 abstracts (see Table 3). Of these, 678/797 (85%; 95% CI, 83–88) provided a trial register name. Authors of RCTs tended to provide the name of a trial register more often compared with other study designs; 312/336 (93%; 95% CI, 90–96) abstracts describing RCTs included the name of an approved trial register compared with 366/461 (79%; 95% CI, 76–83) abstracts describing non-RCTs (e.g., nonrandomized controlled clinical trials, uncontrolled clinical trials, cohort studies). We observed an increase over time in the proportion of authors providing a trial register name from 2007 to 2009 (2007, 74% (191/258; 95% CI, 69–79); 2008, 88% (221/252; 95% CI, 84–92); and 2009, 93% (266/287; 95% CI, 90–96). Authors of about half of all abstracts with a trial register name described an RCT (312/678; 46%, 95% CI, 42–50) and this pattern was consistent across all years.

**Table 3:** Information provided in registration box by authors, compared by RCT status.

Information provided in registration box 2007–2009	RCTs	Non-RCT	Total
	No. (%)	No. (%)	No. (%)
Trial register name	312 (92.9)	366 (79.4)	678 (85.1)
Ethics board	5 (1.5)	24 (5.2)	29 (3.6)
Local authority	2 (0.6)	32 (6.9)	34 (4.3)
Regulatory agency	3 (0.8)	4 (0.9)	7 (0.9)
Pending or reason for no registration	1 (0.3)	0 (0)	1 (0.1)
Other	13 (3.9)	35 (7.6)	48 (6.0)
**Total**	**336 (100)**	**461 (100)**	**797 (100)**

Almost all RCT authors who provided a trial register name specified ClinicalTrials.gov (see Table 4). When we checked the trial registration number provided by the author for RCTs registered in ClincalTrials.gov, we found a valid number for 95% of RCTs (261/276; 95% CI, 92–97). Invalid numbers included reports that registration was pending (n  = 7), or a number that yielded no results upon searching the ClinicalTrials.gov database or was clearly not a valid registration number (n  = 8) (e.g., “0.24”, “1209/07”, “456–07”, “888888”). If one assumes that the numbers reported for registers other than ClinicalTrials.gov are valid, then information included in a trial register could be accessed for 48% (83/172) (95% CI, 41–56) of all ARVO abstracts describing RCTs in 2007, 63% (96/153) (95% CI, 55–70) in 2008, and 69% in 2009 (118/171) (95% CI, 62–76).

**Table 4:** Trial register names provided by authors for randomized trials by year.

Trial register name	2007	2008	2009	Total
	No. (%)	No. (%)	No. (%)	No. (%)
Reference standard	172 (100)	153 (100)	171 (100)	496 (100)
ClinicalTrials.gov(valid number)	74 (43)	86 (56)	101 (59)	261 (53)
ISRCTN	4 (2)	4 (3)	13 (8)	21 (4)
EudraCT	2 (1)	2(1)	1 (0.6)	5 (1)
ANZCTR	1 (0.6)	2 91)	2 (1)	5 (1)
Trialregister.nl	0	0	1 (0.6)	1 (0)
Umin.ac.jp	0	2 (1)	0	2 (0.5)
UK national research register	1 (0.6)	0	0	1 (0)
European clinical trials database	1 (0.6)	0	0	1 (0)
**Total**	**83 (48)**	**96 (63)**	**118 (69)**	**297 (60)**

Abbreviations used: ISRCTN: International Standard Randomised Controlled Trial Number Register, EudrACT: European Union Drug Regulating Authorities Clinical Trials, ANZCTR: Australian New Zealand Clinical Trials Registry, Trialregister.nl: Nederlands Trial Register, Umin.ac.jp: University Hospital Medical Information Network.

## Discussion

We were disappointed that asking authors to identify their abstracts as describing trials is not reliable, since it could have helped to avoid laborious handsearching of conference abstracts required to identify all RCTs for systematic reviews [14]. If we depended solely on author classification of abstracts, we would not have identified a large proportion of trials since authors correctly identified only 75% of RCTs in our reference standard.

One of the reasons for the development of trial registers is to provide information about all initiated trials, including those remaining unpublished. Although registration does inform the public about the existence of trials, most investigations to date have demonstrated that registration details (e.g., study design, protocol and contact information) are less than optimal for inclusion in systematic reviews [10], [15], [16], [17]. Our findings might suggest that a significant proportion - about one third of RCTs (32% [160/496] [95% CI, 28–36]) - reported at ARVO 2007–2009 were not registered publicly. This may be because authors did not consider their study eligible for registration. One possible explanation for why RCT investigators did not register their study is they do not recognize study designs, including those that require registration. For example, a recent study in China found that a high proportion of authors stating in their article that their trial was randomized revealed that they did not fully understand the principles of randomization when queried directly [18]. Our own experience showed that authors of 9/40 ARVO and American Academy of Ophthalmology 1988–89 meeting conference abstracts, who reported use of randomization, responded that assignment to treatment groups was not random when asked directly [19]. Similarly, investigators of other study designs (e.g., non-randomized trial) may not recognize their study design or that it requires registration.

Alternatively, authors may have registered their trials, but may not have entered the required trial registration information on the abstract submission form. Lack of compliance with abstract submission requirements related to trial registration would not be surprising, as compliance with required trial registration is also problematic for journal articles [20], [21]. If including trial registration information is to be meaningful, it should be accurate and complete. Journal editors and conference organizers may wish to require that authors receive formal registration instruction and may need to monitor the submission process more closely.

The fact that authors do not always recognize their trials as RCTs and the apparent lack of trial registration has broad implications for identifying all the evidence for informing systematic reviews and healthcare decision making. If authors have no intention of publishing their findings and registration is not required by law [22] or a research ethics review board, or as a condition of funding by an agency such as the National Institutes of Health [23], there is little incentive to register a trial. Community and commercial research ethics review boards in the US may not require registration [24]. Consistency in requiring trial registration through legislation, across funding and regulatory agencies, and research ethics boards, would likely increase registration and benefit the public and systematic reviewers alike.

### Limitations

Our findings are limited to abstracts submitted to a single conference from 2004 through 2009 and may not apply to other years or areas of clinical research. The relatively low rate of correct identification of RCTs across the years searched implies that there is an ongoing problem with author classification of RCTs. Although we observed a high “false positive” rate for trial registration, this result may be due to the fact that registration is required for many types of “clinical trials” not just RCTs. We did not expect all abstracts with trial registration to be RCTs, but we did expect that all RCTs would be registered. We had hoped that a set of abstracts with trial registration would provide us an enriched and comprehensive source of RCTs to reduce the time and effort required to search the conference abstracts.

### Implications

Our findings lead us to be somewhat pessimistic about authors being able to identify their own studies as randomized clinical trials. In addition, RCT investigators may not be registering their trials or reporting trial registration. Thus, it is unlikely that systematic reviewers would be able to use the author-classification of study design to identify ARVO abstracts describing RCTs.

## References

[1] Scherer RW, Langenberg P, von Elm E (2005) Full publication of results initially presented in abstracts. The Cochrane Database of Reviews. Issue 2. Art. No.: MR000005. doi: 10.1002/14651858.MR000005.pub2 10.1002/14651858.MR000005.pub3 17443628

[2] Chalmers T (1977) Randomize the first patient! [letter] N Engl J Med. 296: 107. 10.1056/NEJM197701132960214 318593

[3] Simes RJ (1986) Publication bias: The case for an international registry of clinical trials. J Clin Oncol 4: 1529–1541. 3760920 10.1200/JCO.1986.4.10.1529

[4] Dickersin K, Rennie D (2003) Registering clinical trials. JAMA 290: 516–23. 12876095 10.1001/jama.290.4.516

[5] Abbasi K (2004) Compulsory registration of clinical trials. BMJ 329: 637–8 doi: 10.1136/bmj.329.7467.637. 15374895 10.1136/bmj.329.7467.637 PMC517630

[6] Ad Hoc Working Party of the International Collaborative Group on Clinical Trial Registries (1993) Position paper and consensus recommendations on clinical trial registries. Clin Trials Metaanal 28: 255–66. 10146333

[7] DeAngelis CD, Drazen JM, Frizelle FA, Haug C, Hoey J, et al. (2004) International Committee of Medical Journal Editors. Clinical trial registration: a statement from the International Committee of Medical Journal Editors. JAMA. 292: 1363–4. 10.1001/jama.292.11.1363 15355936

[8] Krleža-Jerić K, Chan AW, Dickersin K, Sim I, Grimshaw J, et al. (2005) Principles for international registration of protocol information and results from human trials of health related interventions: Ottawa statement (part 1). BMJ 330: 956–8. 15845980 10.1136/bmj.330.7497.956 PMC556346

[9] Gülmezoglu AM, Pang T, Horton R, Dickersin K (2005) WHO facilitates international collaboration in setting standards for clinical trial registration. Lancet 365: 1829–31. 15924966 10.1016/S0140-6736(05)66589-0

[10] Viergever RF, Ghersi D (2001) The quality of registration of clinical trials. PLoS ONE 2011;..6 (2): e14701, doi: 10.1371/journal.pone.0014701 10.1371/journal.pone.0014701 PMC3044717 21383991

[11] Sieving P, Scherer R, Dickersin K (2004) Author self-classification of conference proceeding abstracts [abstract] [Internet]. 12^th^ Cochrane Colloquium, Ottawa, 2–6 October 2004. Cochrane Collaboration website. Available:http://www.imbi.uni-freiburg.de/OJS/cca/index.php?journal=cca&page=article&op=view&path%5B%5D=2743. Accessed 2012 May 30.

[12] Association for Research in Vision and Ophthalmology Statement on Registering Clinical Trials [Internet]. Association for Research in Vision and Ophthalmology website. Available: http://www.arvo.org/EWEB/DynamicPage.aspx?site=AM_2007&WebCode=AbSubGuidelines. Accessed 2012 May 30.

[13] Training Manual for Handsearchers [Internet]. US Cochrane Center website. Available: http://us.cochrane.org/sites/us.cochrane.org/files/uploads/pdf/Handsearcher%20Training%20Manual.pdf. Accessed 2012 May 30.

[14] Institute of Medicine of the National Academies, Committee on Reviewing Evidence to Identify Highly Effective Clinical Services, Board on Health Care Services (2008) Knowing what works in health care: A roadmap for the nation. Washington DC: The National Academies Press, 256 p.

[15] Ross JS, Mulvey GK, Hines EM, Nissen SE, Krumholz HM (2009) Trial publication after registration in ClinicalTrials.Gov: A cross-sectional analysis. PloS Med 6(9): e1000144 doi:10.1371/journal.pmed.1000144. 19901971 10.1371/journal.pmed.1000144 PMC2728480

[16] Moja LP, Moschetti I, Nurbhai M, Compagnoni A, Liberati A, et al. (2009) Compliance of clinical trial registries with the World Health Organization minimum data set: a survey. Trials 10: 56 doi:10.1186/1745-6215-10-56. 19624821 10.1186/1745-6215-10-56 PMC2734552

[17] Liu X, Li Y, Yu X, Feng J, Zhong X, et al. (2009) Assessment of registration quality of trials sponsored by China. J Evid Based Med 2009 2: 8–18 doi:10.1111/j:176-5391.209.1007.x. 10.1111/j.1756-5391.2009.01007.x 21348977

[18] Wu T, Li Y, Bian Z, Liu G, Moher D (2009) Randomized trials published in some Chinese journals: how many are randomized? Trials 10: 46 doi:10.1186/1745-6215-10-46. 19573242 10.1186/1745-6215-10-46 PMC2716312

[19] Scherer RW, Dickersin K, Langenberg P (1994) Full publication of results initially presented in abstracts: A meta-analysis. JAMA 272:158–62. Erratum JAMA1994 272: 1410. 8015133

[20] Reveiz L, Cortés-Jofré M, Asenjo-Lobos C, Nicita G, Ciapponi A, et al. (2010) Influence of trial registration on reporting quality of randomized trials: study from highest ranked journals. J Clin Epidemiol 63: 1216–22. 20430576 10.1016/j.jclinepi.2010.01.013

[21] McGee RG, Su M, Kelly PJ, Higgins GY, Craig JC, et al. (2011) Trial registration and declaration of registration by authors of randomized controlled trials. Transplantion 92: 1094–1100. 10.1097/TP.0b013e318232baf2 21978995

[22] The Food and Drug Administration Amendment Act. U.S. Government Printing Office website. Available: http://frwebgate.access.gpo.gov/cgi-bin/getdoc.cgi?dbname=110_cong_public_laws&docid=f:publ085.110.pdf. Accessed 2012 May 30.

[23] Clinical Trials Registration in ClinicalTrials.gov (Public Law 110–85): Competing Applications and Non-Competing Progress Reports [Internet]. U.S. Department of Health and Human Services website. Available: http://grants.nih.gov/grants/guide/notice-files/not-od-08-023.html. Accessed 2011 July 20.

[24] Western Institutional Review Board website. Available: http://www.wirb.com/. Accessed 2012 May 30.

